# Management of Metabolic Health Syndrome: A Pilot Study in the Early Intervention Service

**DOI:** 10.1192/bjo.2025.10349

**Published:** 2025-06-20

**Authors:** Elishba Chacko, Reem Abed

**Affiliations:** SHSC, Sheffield, United Kingdom

## Abstract

**Aims:** Educating patients about metabolic side effects.

Empowering patients with knowledge and skills to make informed lifestyle choices.

Implementing personalised lifestyle interventions to improve metabolic health parameters.

Monitoring progress to facilitate long-term adherence to healthy behaviours.

**Methods:** Patients between 18–65 years.

Symptoms of metabolic syndrome i.e. high blood pressure, low HDL, truncal obesity, high triglycerides, impaired fasting glucose.

Patients currently/historically on antipsychotic medication.

Patients who have at least a year left in the service were included in the pilot.

Relative stability in mental health i.e. ability to engage with physical health appointments.

**Results:** The pilot concluded that patients benefited from tailored lifestyle interventions, giving them a sense of purpose and accountability.

There were significant changes in waist/circumference ratio; with noted improvement. Waist mean change = −5 cm (−6%); Waist:height mean change =−0.03 (−6%).

There were significant changes in weight: 5 individuals lost weight and improved their BMI; 2 individuals improved from overweight >normal.

There were no significant changes in biochemical markers.

A larger sample is required for a longer duration to study the impact of lifestyle interventions.

**Conclusion:** Small sample size – risk of bias, limited generalizability. Measurements for waist circumference might be prone to error as there is variation in the method of measurement i.e. over or under clothing. Blood results were inconclusive, perhaps the focus of the second phase should be waist circumference since there was marked change and measurable.

Clients would benefit from maintaining a food/mood diary and attending a briefing group at the start of the study to understand the basic principles of nutrition and the digestion process.

